# Offline rTMS inhibition of the right dorsolateral prefrontal cortex impairs reappraisal efficacy

**DOI:** 10.1038/s41598-022-24629-0

**Published:** 2022-12-10

**Authors:** Miroslaw Wyczesany, Agnieszka K. Adamczyk, Justyna Hobot, Giansalvo Barbalinardo, Przemysław Adamczyk, Adam Labaza, Tomasz S. Ligeza

**Affiliations:** 1 Institute of Psychology, Jagiellonian University, Ingardena 6, Kraków, Poland; 2 Behavioural Science Institute, Radboud University, Nijmegen, The Netherlands; 3 Center of Functionally Integrative Neuroscience, Aarhus University, Aarhus, Denmark

**Keywords:** Prefrontal cortex, Attention, Psychology

## Abstract

In this study we verified the causal role of the bilateral dorsolateral prefrontal cortex (DLPFC) in emotional regulation using a strategy of reappraisal, which involves intentionally changing the meaning of an affective event to reduce its emotional impact. Healthy participants (n = 26; mean age = 25.4) underwent three sessions of inhibitory continuous theta burst stimulation (cTBS) applied on three different days over the left or right DLPFC, or the vertex. After applying the stimulation protocol participants were presented with neutral and negative pictorial stimuli that had to be either passively watched or reappraised. The efficacy of emotional control was quantified using the Late Positive Potential (LPP), the neural marker of motivated attention and elaborated stimulus processing. The results showed that reappraisal was compromised after inhibitory stimulation of the right DLPFC compared to the vertex. This impairment of affective modulation was reflected in both early (350–750 ms) and late (750–1500 ms) time windows. As no session differences during the passive watching conditions were found, the decrease in reappraisal efficacy due to non-specific changes in basic perceptual processing was considered unlikely. Instead, we suggest that inhibition of the right DLPFC primarily affects the top-down mechanism of attentional deployment. This results in disturbances of attentional processes that are necessary to thoroughly elaborate the content of affective stimuli to enable their new, less negative interpretation.

## Introduction

Emotion regulation (ER), the ability to effectively regulate undesirable affective states, is an important aspect of adaptive human behavior^1^. ER can be achieved with the use of various behavioral and cognitive strategies that can affect the intensity, duration or quality of emotion^2^. Cognitive reappraisal, a strategy that involves reinterpreting the affective meaning of the stimulus, is considered one of the most effective^3,4^. It is typically used to down-regulate emotional responses to negative stimuli. In consequence, the intensity of negative affect is decreased along with the electrophysiological and peripheral markers of emotional processing^5,6^.

It is commonly agreed that modulating emotional responses using a wide range of ER strategies is initiated and controlled by the prefrontal cortex (PFC)^2,4,5,7^. Indeed, implementing both voluntary and automatic forms of reappraisal increases activity in several brain areas that can be considered parts of the ER network, most consistently the dorsolateral (DLPFC) and the ventrolateral PFC (VLPFC), but the involvement of other brain regions, including the supplementary motor area (SMA), the dorsomedial PFC (DMPFC), and the anterior cingulate cortex (ACC) is also often reported^8–10^. The increased DLPFC/VLPFC activations during efficient implementation of reappraisal tend to be negatively correlated with the activity of the amygdala^11,12^, but also with the ventral striatum, and the left anterior insula — brain regions showing enhanced activity in response to emotional versus neutral stimuli^5,12,13^. Similarly, decreased activations associated with reappraisal are observed in sensory cortices, which indicates that ER already affects early, sensory and attentional stages of information processing^14–16^. Emotional self-report scores, including unpleasantness and arousal in response to emotion-eliciting stimuli, show a negative correlation with PFC activity^10^. Importantly, among patients with affective disorders, the patterns of activations described above are often disturbed, which indicates impairment or inefficient recruitment of regulatory mechanisms^17,18^.

Existing data suggest that the DLPFC's contribution to reappraisal covers multiple cognitive domains. Directing attention to relevant information and maintaining and manipulating strategy-relevant content held in working memory (WM) are considered especially important during the first phase of reappraisal, when a new interpretation is generated^2,7,19,20^. Other cognitive control functions, which are essential to successful ER, are also attributed to the DLPFC^3,21,22^. Action monitoring is necessary to initiate modulatory actions, maintain regulatory goals, and supervise the whole reappraisal process^8,9,23,24^. Last but not least, the DLPFC is claimed to be a source of multidimensional top-down influences (both direct or indirect) towards numerous brain regions, including cortical (prefrontal, anterior cingulate, posterior attentional and visual regions)^25^ and subcortical (amygdala, ventral striatum) areas^13,26–29^.

It is thus not surprising that the DLPFC is widely acknowledged as playing a key role in reappraisal^30^. However, the evidence to support this claim is mostly correlational and direct causal support can hardly be provided. Important methods that allow inferences on causal influences of brain regions are transcranial magnetic stimulation (TMS) and transcranial direct current (electric) stimulation (tDCS), both of which can either enhance or inhibit the activity of a selected region for minutes to hours following a single administration^31^. Only a few reports involving non-invasive brain stimulation have been published in which the effects of DLPFC stimulation on reappraisal efficacy were assessed. In one of these studies two targets were selected, one in the DLPFC and one in the VLPFC, to examine the impact of tDCS on reappraisal of negative pictures^32^. However, contrary to expectations, the DLPFC stimulation showed no effect compared to the sham condition. Only VLPFC stimulation resulted in a decrease in self-reported unpleasantness of reappraised emotional pictures along with a reduction of cardiac interbeat interval events—a measure suggesting increased cognitive engagement during reappraising. In another experiment, the regions within the right DLPFC and VLPFC were stimulated using a 10 Hz excitatory repetitive TMS (rTMS) protocol before participants were asked to use reappraisal^33^. The modulatory effects were quantified using self-report of emotional experience and an event-related EEG measure—the late positive potential (LPP), which reflects the actual emotional arousal^34^. Stimulation of either of these areas brought an increase in reappraisal efficacy compared to vertex stimulation. In another study the anodal (excitatory) tDCS of the right DLPFC resulted in decreased skin-conductance responses and decreased emotional arousal ratings during down-regulation of negative images when compared to the sham condition^35^. Finally, improved reappraisal and cognitive control performance were reported after ten sessions of anodal (excitatory) tDCS to the left DLPFC^36^. However, these results should be interpreted with caution as the effects were based on self-reports only and were limited to a group of bipolar disorder patients. On the one hand, the aforementioned studies are generally consistent with theoretical models that attribute the key role in ER processes to the PFC and supplement them with some causal argumentation. On the other hand, they come with some limitations that still need to be addressed, like the specific groups that were recruited^36^ or the application of bi-hemispheric stimulation, which simultaneously exerts opposite effects on each hemisphere (e.g., excitatory on the right while inhibitory on the left), which makes the interpretation of the findings challenging. The latter issue remains important due to hemispheric differences of the DLPFC in emotional processing^37,38^, which are poorly recognized in ER research.

To address the above limitations, we used a within-study design to examine the causal contribution of the left and right DLPFC in down-regulation of emotional responses using cognitive reappraisal. We applied a TMS protocol, namely continuous theta burst stimulation (cTBS) over either the left or right DLPFC in healthy young adults and observed the effects on the subsequent ER efficacy. We used cTBS because of its focal stimulatory effect and its predominant inhibitory impact on cognitive performance when applied to the DLPFC^39^. To limit the impact of demand characteristics, we used an active control condition, which involved stimulation of the cranial vertex (the highest point of the head). The stimulation took place on three different days, which resulted in three TMS sessions per participant. To measure ER efficacy, we used a classic reappraisal paradigm. In the reappraisal condition participants down-regulated emotions to negative pictures (NEG-REAP); in the passive watching condition, they were asked to respond naturally to negative (NEG-WATCH) and neutral (NEU-WATCH) pictures. This yielded a 3 × 3 design with factors: TMS session (L: left DPFC, R: right DLPFC, V: vertex) and strategy (NEG-REAP, NEG-WATCH, NEU-WATCH). Reappraisal was limited to down-regulation of negative pictures only, as such a condition can be considered more ecologically valid (and clinically relevant) as it is closely related to everyday psychological functioning. To quantify regulatory effects, we applied electroencephalography (EEG) recording and measured the centro-parietal LPP component, a marker of sustained motivated attention and emotional arousal^34^, along with subjective ratings of negative emotional experience in response to the presented pictures. As the LPP covers multiple stages of emotional processing, with the early LPP better reflecting attentional processes and the late LPP more related to semantic elaboration processes^40^, we separated this component into early (LPP1: 350–750 ms) and late (LPP2: 750–1500 ms) time windows^34,41^.

As a manipulation check, we expected to see the typical reappraisal effects after the vertex stimulation, i.e. decreased LPP amplitude and more positive subjective ratings in response to negative stimuli. The main hypothesized effect would be seen as decreased efficacy of reappraisal after cTBS inhibitory stimulation to either the L or R DLPFC compared to the vertex. We expected this deterioration to be reflected in a lack of significant difference in the LPP and subjective rating responses between the NEG-REAP and NEG-WATCH conditions. Finally, as reports of more general alterations in bottom-up perceptual processes after rTMS stimulation of DLPFC are present in the literature^38^, we could not rule out that such a nonspecific influence also affects LPP amplitudes and subjective ratings in passive watching trials. Therefore, such effects were also checked.

## Results

### Behavioral data

#### Manipulation check

As expected, comparison of the NEG-REAP vs. NEG-WATCH conditions in the V session revealed that participants reported less negative emotional state in the reappraisal compared to the watch condition: *t*(22) = − 3.62, *p* = 0.002; *p*_FDR_ = 0.006.

#### The effect of DLPFC inhibition on reappraisal efficacy

Contrary to our predictions, a comparison of differences in valence ratings (NEG-WATCH minus NEG-REAP) revealed no effect of the TMS session, L vs V: *t*(44.3) = 0.60, *p* = 0.55; *p*_*FDR*_ ns; R vs V: *t*(44.1) = − 0.10, *p* = 0.92; *p*_*FDR*_ ns.

#### The effect of TMS session on passive watching trials

**Figure 1:**
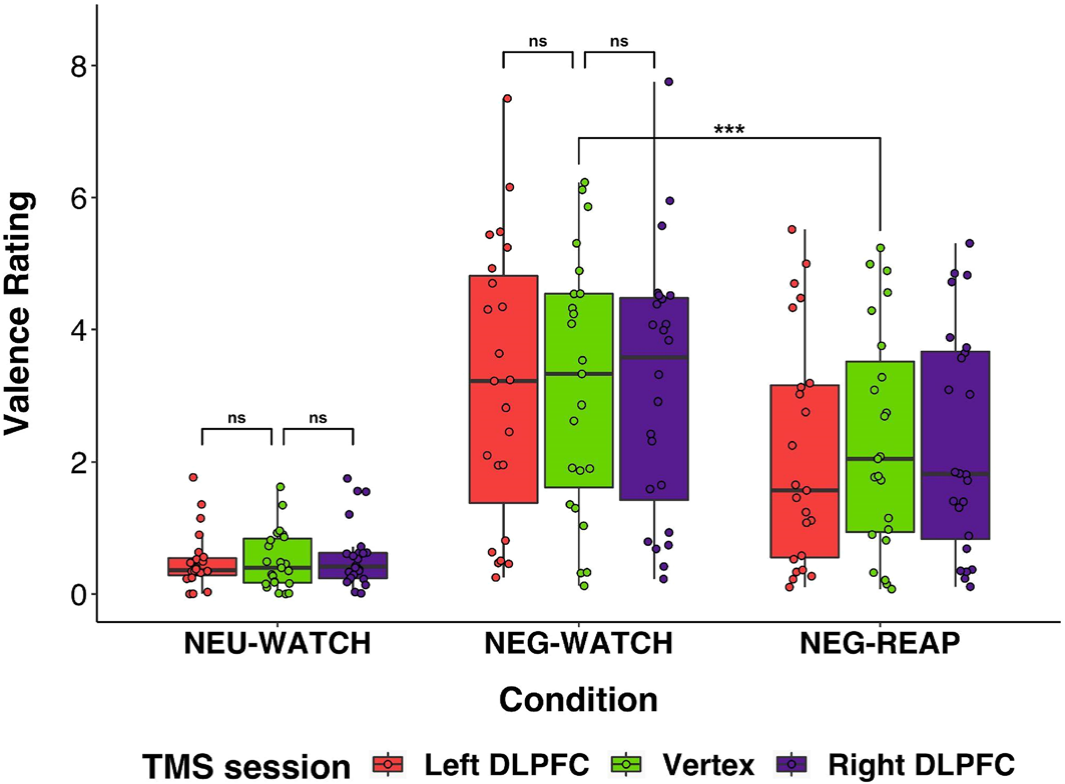
Results of valence ratings. Note. Boxplots for the valence ratings for strategy (NEU-WATCH, NEG-WATCH, NEG-REAP) x TMS session (left DLPFC, vertex, right DLPFC). Black horizontal lines within the box indicate the median, the box indicates the upper and lower quartile limits. Individual results are shown as dots. Valence Rating (0 = neutral, 10 = unpleasant). *NEG-REAP* negative-reappraisal, *NEG-WATCH* negative-watch, *NEU-WATCH* neutral-watch. ***p < *.*001, *ns* non-significant.

Self-reported negative emotions did not differ as a function of session in the NEU-WATCH, L vs V: *t*(43.5) = − 0.04, *p* = 0.97; *p*_*FDR*_ ns; R vs V: *t*(43.4) = 0.34, *p* = 0.74; *p*_*FDR*_ ns, and NEG-WATCH conditions, L vs V: *t*(44.3) = − 0.23, *p* = 0.83; *p*_*FDR*_ ns; R vs V: *t*(44.1) = − 0.36, *p* = 0.72; *p*_*FDR*_ ns. Self-report results are presented in Fig. 1.

### EEG data

#### Manipulation check

Our reappraisal manipulation was shown to be successful as seen in the later time window. This was reflected in a decrease of LPP2 amplitude in NEG-REAP compared to NEG-WATCH trials in the V session over the CPz (*t*(23) = 2.76; *p* = 0.011; *p*_*FDR*_ = 0.036) and Pz electrodes (*t*(23) = − 2.72; *p* = 0.012; *p*_*FDR*_ = 0.036). No significant reappraisal effects were detected in the early LPP1 window.

#### The effect of DLPFC inhibition on reappraisal efficacy

**Figure 2:**
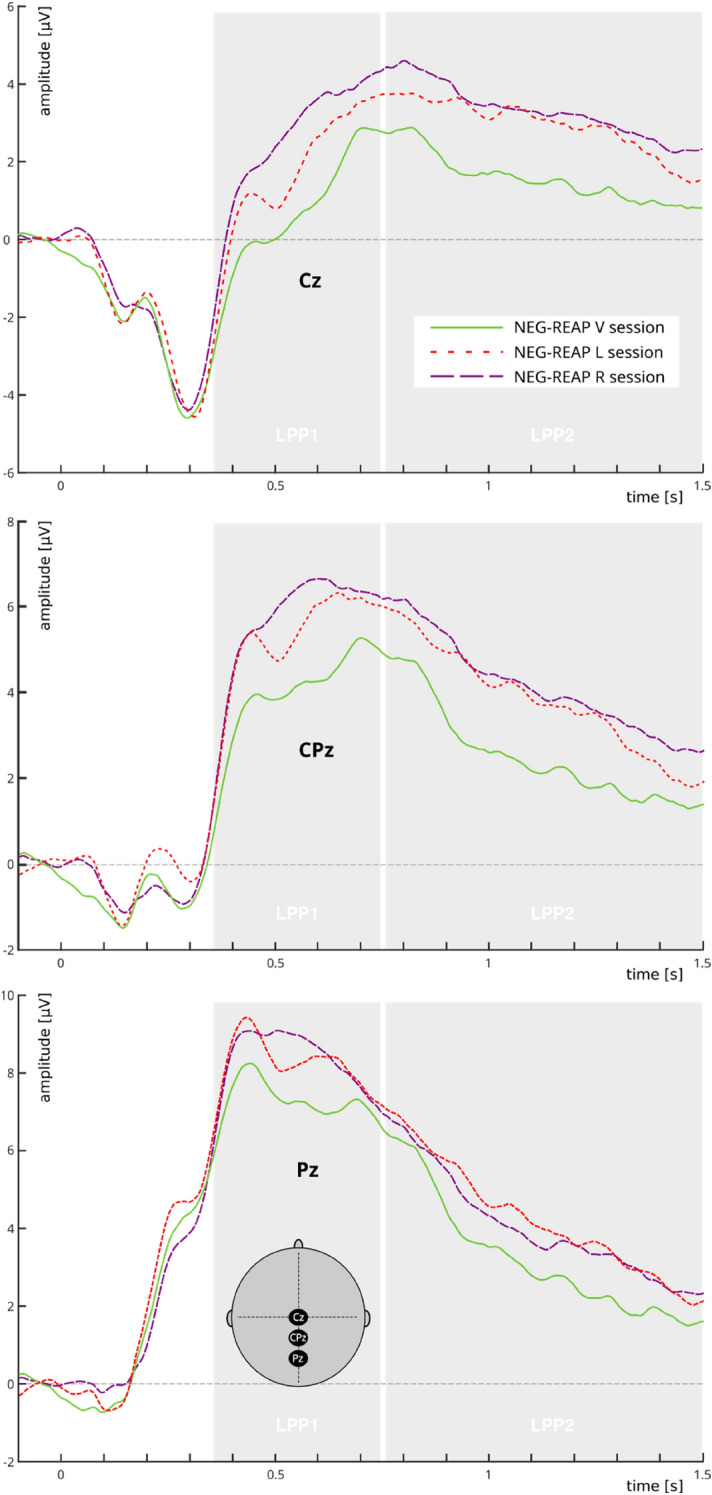
Picture-locked grand average waveforms at electrodes Cz (upper plot), Cpz (middle plot), and Pz (lower plot) for the reappraisal conditions (NEG-REAP) after the three TMS sessions (L, R, V). Shaded gray areas indicate the LPP time-windows submitted to statistical analysis (LPP1: 350–750 ms, LPP2: 750–1500 ms). Time zero on the x-axis indicates picture onset. The x-axis runs from the beginning of the baseline (100 ms prior to picture onset) to 1500 ms after picture onset. The EEG montage for the considered electrodes is included.

**Figure 3:**
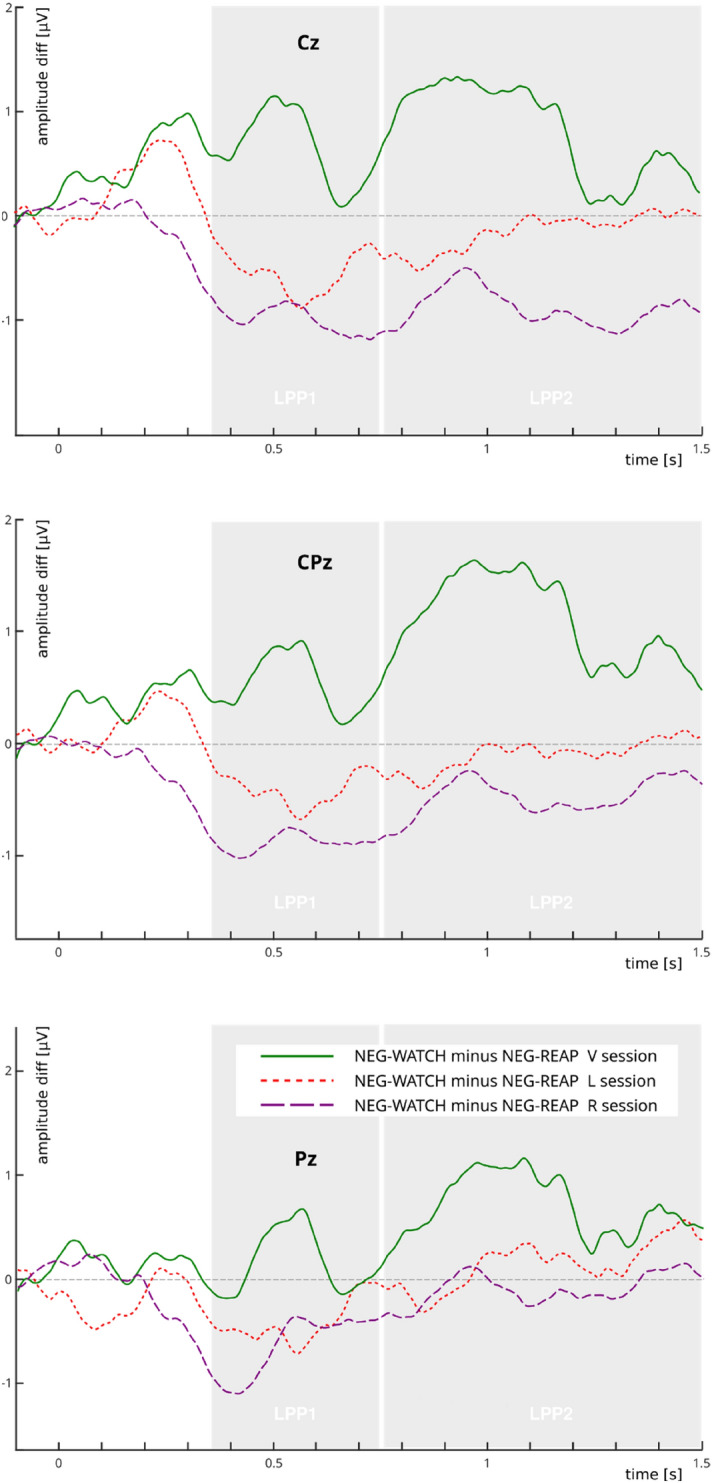
Amplitude differences in grand averages for the NEG-WATCH minus NEG-REAP conditions at electrodes Cz (upper plot), Cpz (middle plot), and Pz (lower plot) after the three TMS sessions (L, R, V). Positive values are associated with a decrease in the LPP amplitude after reappraisal relative to passive watch, which reflects successful emotion downregulation. Shaded gray areas indicate the LPP time-windows submitted to statistical analysis (LPP1: 350–750 ms, LPP2: 750–1500 ms). Time zero on the x-axis indicates picture onset. The x-axis runs from the beginning of the baseline (100 ms prior to picture onset) to 1500 ms after picture onset.

Confirming the main experimental hypothesis, comparison of NEG-WATCH minus NEG-REAP LPP amplitude differences between left/right DLPFC TMS versus vertex TMS sessions demonstrated that disruption of the right DLPFC impaired reappraisal-induced down-regulation of LPP amplitude over the centro-parietal sites for both the LPP1 (Cz: *t*(48.5) = 2.63; *p* = 0.011; *p*_*FDR*_ = 0.023; CPz: *t*(48.0) = 2.62; *p* = 0.011; *p*_*FDR*_ = 0.023) and the LPP2 time windows (Cz: *t*(48.2) = 2.78; *p* = 0.007; *p*_*FDR*_ = 0.023; CPz: *t*(50.0) = 2.44; *p* = 0.018; *p*_*FDR*_ = 0.027). No significant effects were observed for the left DLPFC stimulation. The statistics for the LPP component are shown in Table 1 and the plots are depicted in Figs. 2 and 3.

**Table 1:** Statistics for the LPP component.

Time window	Electrode	*t*	*df*	*p*	*p*_FDR_
**NEG-WATCH—NEG-REAP (V session)**					
LPP1 (350–750 ms)	Cz	0.73	23	0.469	ns
	CPz	1.58	23	0.127	ns
	Pz	1.63	23	0.110	ns
LPP2 (750–1500 ms)	Cz	1.85	23	0.076	ns
	CPz	2.76	23	0.011	0.036
	Pz	2.71	23	0.012	0.036
**NEG-WATCH—NEG-REAP**					
**V vs. L**					
LPP1 (350–750 ms)	Cz	1.52	48.5	0.134	ns
	CPz	1.42	48.0	0.161	ns
	Pz	0.77	47.9	0.440	ns
LPP2 (750–1500 ms)	Cz	1.31	48.2	0.196	ns
	CPz	1.67	50.0	0.101	ns
	Pz	0.79	47.9	0.430	ns
**V vs. R**					
LPP1 (350–750 ms)	Cz	2.63	48.5	0.011	0.023
	CPz	2.62	48.0	0.011	0.023
	Pz	1.23	47.9	0.224	ns
LPP2 (750–1500 ms)	Cz	2.78	48.2	0.007	0.023
	CPz	2.44	50.0	0.018	0.027
	Pz	1.11	47.9	0.270	ns

#### The effect of the TMS session on passive watching trials

Data analysis revealed that TMS did not influence basic perceptual processes as there were no significant effects of the TMS session on the NEG-WATCH or NEU-WATCH waveforms for any considered electrodes and time windows.

## Discussion

The study explored the effects of cTBS delivered to either the left or right DLPFC on reappraisal effectiveness. We intended to verify whether inhibiting these areas impairs affective down-regulation, thus supporting a causal role of the DLPFC in reappraisal strategies.

Firstly, as a manipulation check, reappraisal effects were verified using EEG and self-report data from the control (vertex) session. As expected, during reappraisal of negative images, the amplitude of the LPP component was decreased compared to the passive watching condition. Specifically, the effect was observed over the centro-parietal CPz and Pz derivations in the late window. This down-regulation of electrocortical responses following reappraisal was accompanied by a more positive experience as reported by participants.

Our results partly supported the main experimental hypothesis. The inhibitory stimulation compromised reappraisal compared to the vertex session, but only when delivered to the right DLPFC. This effect was reflected in smaller changes of LPP amplitudes between reappraisal and watch trials. In other words, inhibition of the right DLPFC resulted in impairment of ER, as indicated by reduced LPP attenuation during reappraisal. Interestingly, after the inhibition of the left DLPFC we did not observe significant disruption of reappraisal, as was seen after right-side stimulation. As our expectations stem from the data provided in several meta-analyses that demonstrated left and right DLPFC activations associated with reappraisal, we still cannot rule out the possibility that the effect of the left hemisphere was too weak to be observed as statistically significant. Our results also align with findings which demonstrated the enhancement of reappraisal effects after excitatory rTMS was applied over the right DLPFC; however, only the right side was stimulated in this study^32^. Moreover, some evidence suggests that the right DLPFC is recruited only when the reinterpreted content is cognitively demanding (e.g. because of high-intensity emotions)^42^. Thus, inhibition of the right DLPFC could decrease the overall reappraisal efficiency by reducing the ability to down-regulate particularly intense stimuli. On the other hand, inhibition of the left DLPFC could make the reinterpretation more cognitively demanding but still possible due to the (compensatory) involvement of the right DLPFC. Another speculative explanation of the unilateral, right-side effect that was observed can be based on different roles played by left and right DLPFC in reappraisal. While the right DLPFC subserves inhibition of the imposing stimuli interpretation^43^, the left PFC is more involved in linguistic processes, like generating word labels^44^. Thus, the former function could be more critical than generating a new, semantically complicated interpretation.

According to our initial theoretical considerations, the effect of compromised reappraisal was likely underlain by the disruption of cognitive control functions mediated by the DLPFC. Following this claim, and to rule out the possibility that the effect was merely perceptual, we ran an additional test. It was checked whether the stimulation did not affect ERP and self-report responses in the passive viewing conditions, where the cognitive control functions were not recruited. Such an observation would favor the general impact of the stimulation on perceptual processes that could, in turn, non-specifically affect ER. As the analysis did not confirm any influences in the passive watching conditions, we assumed that emotional perception remained relatively intact. Moreover, as the *difference* between the NEG-WATCH and NEG-REAP conditions was considered during the verification of the main hypothesis, we can strengthen the conclusion that the effect of reappraisal disruption was driven by enhanced emotional processing of negative images after the right DLPFC inhibition.

The high temporal resolution of the EEG recording enables us to comment on specific processes that could be disturbed by the cTBS. To do so, we will now briefly consider the consecutive processes that underlie the successful implementation of reappraisal. One of the prerequisites involves directing attention to important features of the examined scene to understand and semantically elaborate its meaning. After the stimulus is appraised, its initial negative interpretation must be maintained in working memory (WM). This interpretation can be then used as a template for the construction of an alternative more-neutral interpretation of the depicted scene. After the alternative reinterpretation is prepared, it must be implemented and its effectiveness tested. Thus, the following reappraisal stages involve (a potentially iterative process of) implementation and monitoring of the effectiveness of the constructed re-interpretation(s). Finally, if the newly constructed interpretation is efficient in down-regulating negative emotions, the initial interpretation has to be inhibited to complete the reappraisal task successfully.

The session differences emerged in the early LPP1 time window and remained visible in the late LPP2 window. This indicates that the TMS was already able to affect reappraisal at its early stage, marked by the 350–750 ms window. Given this relatively early onset of the session effect, a plausible explanation would point at top-down attentional control as one of the first-affected functions. Our indication is supported by several pieces of evidence. Firstly, DLPFC remains active during both reappraisal and distraction emotional-regulation strategies, both of which involve specific requirements for attentional deployment: while reappraisal requires directing attention toward the affective content, distraction involves diverting attention away from the emotional scene, despite bottom-up attraction by high-arousal content^45^. Secondly, the functional significance of early LPP (up to approximately 1000 ms) is associated with attention allocation, while later time windows are more often linked with memory and reinterpretation stages^46^. Thus, the direct TMS effect could be interpreted as a disturbance in the efficient allocation of top-down attention to the presented stimuli that is necessary to process the presented scene in order to understand its meaning and prepare a new, less negative interpretation. Finally, this interpretation is further supported by converging results of several studies. Firstly, enhanced attentional processing of *task*-*relevant* emotional stimuli was revealed after excitatory stimulation to the right DLPFC^37^. Secondly, our interpretation is also in agreement with the data on the effects of DLPFC stimulation on bottom-up attention, which demonstrated that inhibition of the right (but usually not the left) DLPFC amplifies attentional processing of negative stimuli that are *task*-*irrelevant* (i.e. serve as distractors to another task)^35,45^. Moreover, another study reported specific attentional task-related enhancement of LPP amplitude after right anodal (excitatory) tDCS to the DLPFC, while more general modulation of emotional perception during a passive viewing task was not present^47^.

Although the neural generators of the LPP are still debated, it is plausible to link this ERP component with a widespread activation in the parietal, occipital and temporal cortices in response to significant and arousing stimuli^48^. In line with our attentional interpretation we speculate that the weakening of the top down inhibition from the DLPFC would result in increased activity of multiple brain regions relevant for perceptual and emotional processing and then be reflected in the LPP amplitude over the centro-parietal electrodes. Importance of such widespread influences from the DLPFC during cognitive activity and emotional control was already confirmed using effective connectivity methods^25,49^, and is also supported by the rich structural connectivity of this area^50^. While supported by some experimental data, the attentional sources of reappraisal impairment after DLPFC inhibition should still be treated as an inspiring hypothesis to be tested in future studies.

We can also consider here an alternative interpretation that assumes disturbances of the working memory (WM) processes in our procedure. WM is engaged during both the early and late stages of reappraisal. However, the WM load seemingly increases in the later phase due to the maintenance of a new interpretation that has to neutralize stimulus negativity^47^. Indeed, the relationship between WM efficiency and reappraisal success has been reported^20,51,52^. As our effects of cTBS appeared quite early, the interpretation which indicates the disruption of WM as a source of reappraisal decline seems less plausible. However, taking into account the fact that the DLPFC is a part of the network responsible for WM^53^ and that reappraisal consistently activates brain regions implicated in the WM^5,10^, we encourage future studies to design an experiment that would be optimized to disentangle the unique contribution of this region to reappraisal efficacy.

Despite the observed modulation of electrocortical responses after TMS, we failed to observe the effects of the TMS session at the behavioral level. A possible reason for this discrepancy is that the effects of stimulation could have been too subtle to be detected at the subjective experience level with the experimental task employed. Although the impact of affective down-regulation on self-report is widely reported in typical reappraisal procedures, it is often not observed in paradigms where the modulatory goal is implicit and remains undisclosed. In such cases, similarly to our data, self-report ratings tend not to differ even though brain or physiological markers show attenuation of affective processing^9,54–56^. Additionally, it has been reported that even vertex stimulation used as a control condition can impact the estimation of affective stimuli, which raises further doubts regarding the reliability of the first-person methods in our (and similar) procedures^57^.

Finally, some advantages and possible drawbacks of our design should be discussed. The paradigm used here was based on inhibiting rather than boosting the activation of a single neural area during the performance of a specific cognitive task. As such, our procedure could involve a group of healthy participants, thus allowing for an interpretation of results that is similar to the temporary ‘loss of function’ or lesion studies, which enable a more straightforward interpretation of results^58^. Moreover, contrary to the majority of previous studies, we used a repeated-measures design, which provided us with higher power to detect even subtle stimulation effects. Regarding the limitations, it should be remembered that although TMS allows for the selective disruption of activity in a unilateral brain region, if the specific cognitive function is distributed bilaterally, the remaining neural activity can potentially ‘take over’ and account for the less-pronounced activation in its mirror structure. Furthermore, we used the same pictorial stimuli throughout all three TMS sessions. Although this design choice facilitated between-session comparison and control for both low- and high-level stimulus features, it also ran the risk of observing weaker emotion-induction effects (or stronger emotion-regulation effects) due to repeated exposure to the same images. Due to pragmatic issues, we did not include more than one TMS control condition. It cannot be fully dismissed that the control vertex stimulation could also induce some attention-related or arousal-related effects (e.g.^59^). However, we consider this potential risk as low as we verified that the vertex cTBS did not impact perceptual processes and emotional responding. Moreover, reappraisal in the vertex condition resulted in the typical reduction in the LPP amplitude relative to the passive watching condition. Finally, our choice of TMS targets, although based on meta-analytical works, could not fully cover the spatial extent of the DLPFC areas reported in the reappraisal literature. Thus, we advise future studies to replicate our findings, especially exploring slightly modified TMS targets and different stimuli material. Moreover, the analysis of the DLPFC effective connectivity with other cortical areas and its changes following TMS stimulation seems to be an important future direction.

To conclude, our study offers a novel contribution by demonstrating that stimulation of the right but not the left side of the DLPFC compromises reappraisal, starting from its early stages. We indicate attentional deployment mechanisms as a potential source of these disturbances, however this possibility warrants further investigation. Our findings may have implications for the treatment of affective and anxiety disorders, where the efficient emotional regulation is an important therapeutic target. Successful reappraisal requires proper top-down attentional engagement towards emotional stimuli, which might be disrupted in anxiety disorders due to the hypervigilance followed by later *disengagement* from processing of anxiety-provoking stimuli^60^. Therefore, attention control training, combined with the DLPFC excitatory stimulation to counteract these maladaptive attentional tendencies is a potential investigation direction. Another interesting direction would be to examine individual differences in reappraisal efficacy and their link to the right DLPFC activity, including its connectivity with other brain regions. This would potentially constitute a marker of emotional regulation effectiveness, that could be individually monitored in the course of affective control training or therapeutic interventions.

## Materials and methods

### Participants

The experimental procedure was compliant with the directives of the Helsinki Declaration (1975, revised 2000) and was approved by the Jagiellonian University Institute of Psychology Research Ethics Committee. Informed consent was obtained from all participants. Twenty-six volunteers participated in the study (mean age = 25.4, SD = 3.5; 15 females, 10 males, 1 other gender). A similar sample size was used in previous studies that investigated the effect of rTMS on emotion regulation^33^. Due to a technical issue, the behavioral data of two participants were not recorded. EEG data from two subjects were discarded due to technical malfunctions during recording, while one additional subject was excluded from the EEG analysis because its extreme signal values exceeded the 5 IQR (interquartile range) threshold. Thus, 24 participants were included in the self-reports analyses and 23 in the EEG analyses. The reimbursement fee for participation in the study was €40.

### Pictorial material

A total of 90 pictures were selected from the Nencki Affective Pictures System (NAPS)^61^ and the International Affective Picture System (IAPS)^62^: 30 were neutral (food, landscapes, households, neutral animals, people in daily activities) and 60 were negative (disgusting food, sad people, accidents, violent images, animal mutilations, surgical procedures). We attempted to match unpleasant and neutral stimuli in terms of both color content and complexity (e.g., number of faces, number of body parts, etc.). Normative ratings of the pictures (pulled from normalized NAPS and IAPS values) were as follows: for neutral pictures, mean valence = 4.99; SD = 1.07, mean arousal = 4.94; SD = 1.13; for negative pictures, mean valence = 2.89; SD = 1.29, mean arousal = 6.93; SD = 1.42. The neutral and negative sets differed in their normative ratings of valence (*t*(29) = 50.40, p = 0.001) and arousal (*t*(29) = − 21.57, p = 0.001). Another 21 negative pictures were selected to enable reappraisal training before the experimental session. To facilitate comparisons, the same pictures were used for each TMS session, but it was ensured that negative stimuli were always associated with the same (either WATCH or REAP) instruction.

### Experimental design

**Figure 4:**
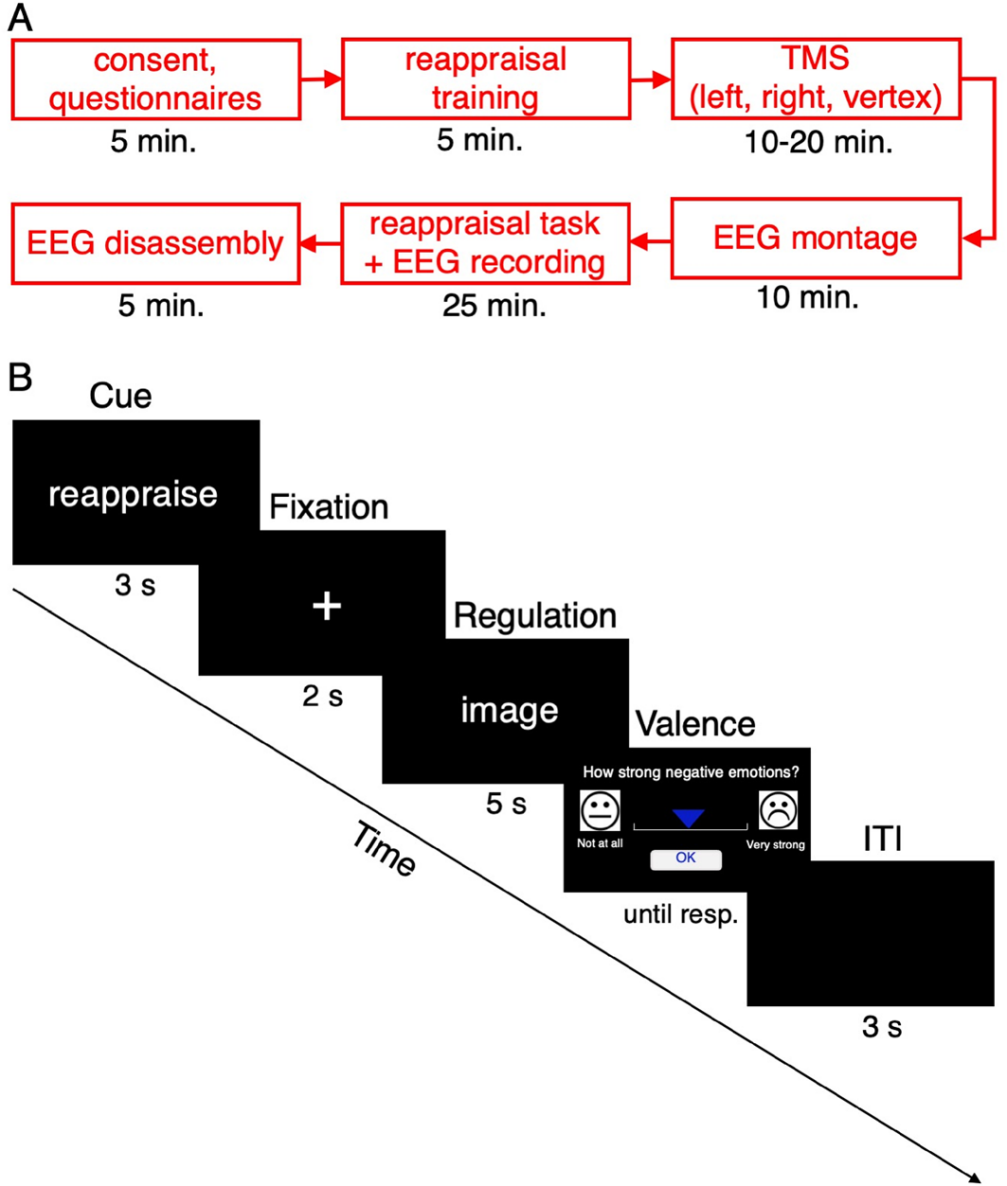
The experimental procedure. (**A**) Overview of a single experimental session. (**B**) Sample trial sequence. *ITI* inter-trial interval.

An overview of an experimental session is provided in Fig. 4. The study applied a reappraisal paradigm and stimuli presentation timing similar to previous studies^63^. All except one participant underwent three rTMS sessions, which were carried out on different days at possibly similar time of the day in counterbalanced order, with either left DLPFC, right DLPFC or control (vertex) stimulation (further referred to as L, R, V sessions, respectively). A single experimental session lasted for ~ 60–80 min, of which ~ 25 min was the experimental task itself (the first session was longer, as it included determination of individual RMT; see TMS procedure). Participants were advised to have enough sleep in the preceding night and refrain from consuming any stimulants before the sessions. Upon arrival, a brief description of the experimental procedure was provided and participants were asked to sign an informed consent form and complete a TMS safety questionnaire. Before the initial experimental session, participants underwent reappraisal training. As reappraisal is an attention-engagement strategy^64^, we used situation-focused reappraisal to distinguish it from other strategies that involve attention disengagement^63,65^. Using sample stimuli, participants were taught to find and apply less negative interpretations of emotional scenes by imagining more positive outcomes of the depicted situation. The participants’ ability to reappraise according to the instructions was verified by the experimenter. Following EEG cap montage (with no electrodes) but before the experimental task, participants underwent a cTBS session. Next, the EEG electrodes were attached. The task was presented in full-screen mode (62 cm diameter LED screen, viewing distance of 60 cm approx.). The task consisted of 90 trials (30 trials per condition) in a pseudo-randomized order, with a half-minute break after the 30th and 60th presentation. Each trial started with the presentation of a cue (3 s) that could be either 'WATCH' or 'REAPPRAISE', which meant passively watching pictures vs using the previously trained reappraisal strategy, respectively. Then a fixation cross was shown (2 s), followed by a picture (2.5 s). Trials concluded with the subjective rating of the negative affective experience on a continuous visual analogue scale (until response); this was followed by a blank screen before the next trial (3 s) (Fig. 3B). Summarizing the design of the experiment, there were 3 conditions, differing with picture valence and instruction cue, i.e. NEU-WATCH (neutral picture, passive watching), NEG-WATCH (negative picture, passive watching), NEG-REAP (negative picture, reappraisal instruction) and 3 TMS sessions (L, R, V).

### TMS procedure

**Figure 5:**
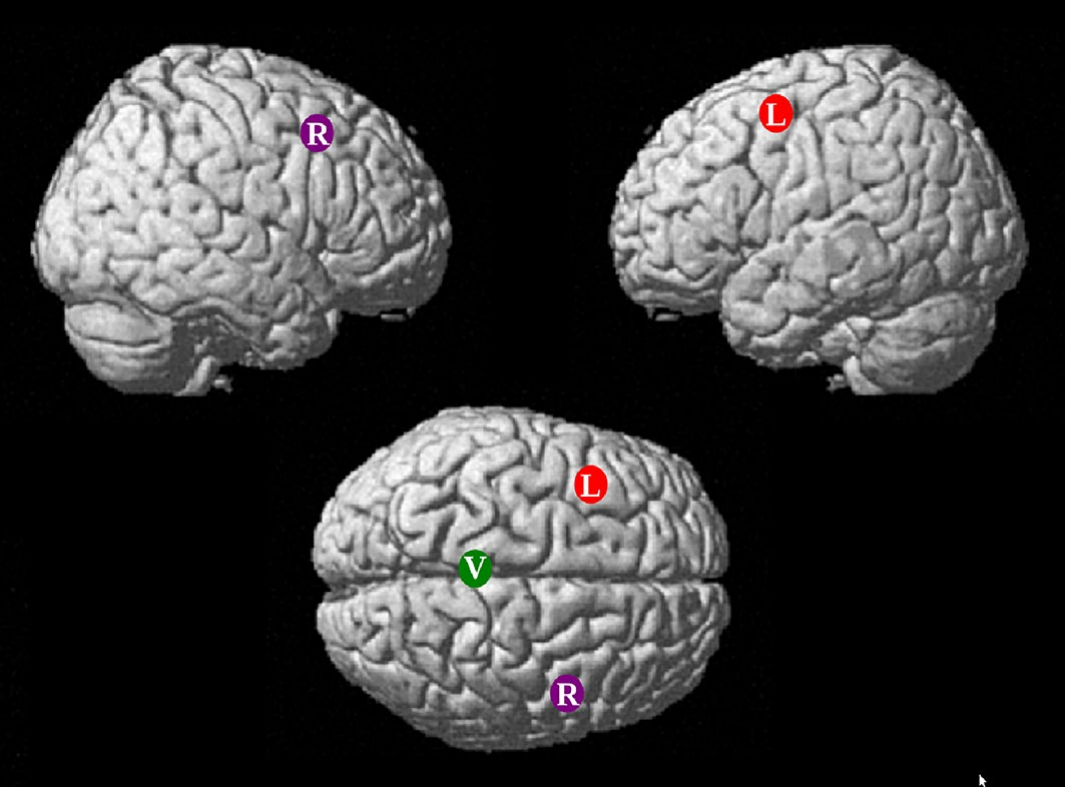
TMS targets for all three sessions (L, R, V) presented on a standard brain surface. Brain surface provided by the SPM12 software.

Biphasic TMS (~ 400 µs) was delivered with a Magstim Super Rapid2 Plus1 stimulator using a 70 mm figure-of-eight air-cooled Double Air Film Coil. For the purpose of resting motor threshold (RMT) estimation, the electromyographic signal was recorded with a Brainsight unit from the first dorsal interosseous (FDI) muscle of the right index finger during the motor threshold (MT) estimation. In the first experimental session, the individual RMT was estimated. Stimulation started from applying 30% of the maximum stimulator output (MSO) single-pulse TMS to the left primary motor cortex. Then, by varying the stimulation intensity, the site where the suprathreshold TMS induced the maximal twitch in the right index FDI muscle was established. Subsequently, the lowest intensity that resulted in a motor-evoked potential (MEP) of more than 50 μV peak-to-peak amplitude in 5 out of 10 consecutive trials was determined. The stimulation sites were localized with the Brainsight 2.3 neuronavigation software (Rogue Research Inc.) using individual structural MRI scans. Each participant’s brain was transformed into the standard Montreal Neurosciences Institute (MNI) space using Brainsight software. Depending on the session, TMS was applied to either the left DLPFC (middle frontal gyrus; − 33, 3, 54; L session), the right DLPFC (middle frontal gyrus; MNI coordinates: 51, 15, 48; R session), or the vertex (0, − 28, 90; V session), with the coil handle pointing backward (see Fig. 5). The DLPFC stimulation sites were selected based on reappraisal meta-analysis^5^. The site of stimulation and the tangential position of the coil in relation to the scalp were ensured by using the neuronavigation system. Throughout the RMT determination procedure and subsequent application of cTBS to the DLPFC, the coil was held at a 45° angle off the midline, with the handle pointing down, while for the vertex stimulation the coil remained untitled. The current induced in the brain was PA–AP. During the cTBS stimulation, participants kept their eyes open. The cTBS protocol had a classic pattern and duration, i.e. 3-pulse bursts at 50 Hz were applied at 5 Hz for 40 s^66^. The cTBS was delivered at 80% of the individual RMT and the average intensity equaled 61.1% (SD = 12.2) of the MSO. Participants wore earplugs for noise protection throughout the duration of TMS.

### EEG recording and analysis

The EEG signal was recorded using a BiosemiTwo device and a set of 64 cap electrodes, supplemented by two mastoid and four oculomotor electrodes. Data from two participants had to be discarded due to technical problems that arose during the procedure. The EEG signal was processed using FieldTrip-based custom routines (Atlantis processing toolbox; http://atlantis.psychologia.uj.edu.pl). The following steps were applied: reference to the linked mastoid; 0.2 Hz high-pass and 46 Hz low-pass filtering; bad channel screening using trimmed variance extreme values; eye-movement correction with the recursive least square (RLS) method^67^; segmentation relative to stimulus onset in the -0.1 to 2.5 s window; baseline correction using the prestimulus period; either interpolation of bad channels or whole-trial rejection (depending on the number of electrodes affected). Range (100 µV threshold), excess variance or muscle signal contamination (detected by spectral power value for frequencies > 35 Hz) artifacts were finally removed, and the remaining trials (78.9 on average) were averaged across conditions. LPP magnitude was quantified as the average value within the following time windows (LPP1: 350–750 ms, LPP2: 750–1500 ms), which were selected on the basis of the literature^41^. As the LPP is maximal over centro-parietal electrodes^48^, mean LPP amplitudes were measured from electrodes Cz, CPz, Pz^68,69^.

### Statistical analysis

The statistical analyses were performed by fitting a series of linear mixed-effects (LME) models using the *lme4* package, and the *lmerTest* library was used to estimate related p-values^70^. Each model included condition as a fixed effect and a participant-specific intercept. The resulting p-values were subjected to additional False Discovery Rate (FDR) correction together for all the three electrodes and two time windows considered. LME modeling was used to account for missing data, while the *lmerTest* package applied the Satterthwaite method for estimating degrees of freedom.

As a manipulation check for emotional regulation, we compared NEG-REAP vs. NEG-WATCH data in the V session, which was considered a session without any DLPFC stimulation. To further verify that the TMS stimulation did not alter perceptual processes per se, which would affect the interpretation of the results, we also determined the effect of the stimulatory session on the NEU-WATCH waveforms. Moreover, to determine whether emotional responding itself could be affected by stimulation, we checked the effect of the TMS session on NEG-WATCH LPP amplitudes, which reflects the overall amount of resources captured by the processing of emotional stimuli and the intensity of emotional response. Crucially, to verify that the TMS affected emotion regulation, we compared the session effect on the difference between the NEG-REAP and the NEG-WATCH conditions. We considered the differences between the watch and reappraisal conditions instead of absolute LPP amplitudes to minimize the possible confounding effects of more general TMS-induced alterations of perceptual or emotional processing that could still be present, even when not detected by previously described manipulation checks.

## Data Availability

The datasets used and/or analyzed during the current study available from the corresponding author on reasonable request.
